# A novel technique to harvest bone autografts with mild local hyperthermia and enhanced osteogenic bone quality: a preclinical study in dogs

**DOI:** 10.1186/s12903-023-03611-w

**Published:** 2023-11-07

**Authors:** Tengfei Zhou, Zekun Gan, Hanfei Zhang, Ziyi Liu, Yiping Pu, Mingdeng Rong

**Affiliations:** 1 Department of Periodontology and Oral Implantology, Stomatological Hospital, Southern Medical University, Guangzhou, 510280 China; 2 Stomatological Hospital, Southern Medical University, Guangzhou, 510280 China; 3 Department of Oral Surgery, Shanghai Ninth People’s Hospital, College of Stomatology, Shanghai Jiao Tong University School of Medicine, Shanghai, 200001 China

**Keywords:** Bone autografts, Guided bone regeneration, Osteotomy, Bone drilling, Bone temperature, Thermal exposure

## Abstract

**Background:**

Guided bone regeneration (GBR) involves collecting bone autografts with high bio-quality and efficiency. The current non-irrigated low-speed drilling has been limited for broader application in bone autograft harvest due to its low efficiency, inability to conduct buccal cortical perforation, and dependence on simultaneous implant placement. Increasing the drilling speed helps improve the efficiency but may incur thermal-mechanical bone damage. Most studies have addressed thermal reactions during bone drilling on non-vital models, which is irrelevant to clinical scenarios. Little has been known about bone’s in vivo thermal profiles under non-irrigated higher-speed drilling and its influences on the resulting bone chips.

**Aim:**

A novel technique for bone harvest and cortical perforation via in-situ non-irrigated higher-speed drilling was proposed and investigated for the first time.

**Methods:**

The third mandible premolars of eight beagles were extracted and healed for three months. Sixteen partial edentulous sites (left and right) were randomized into four groups for bone autograft harvest without irrigation: chisel, 50 rpm drilling, 500 rpm drilling, and 1000 rpm drilling. Bone chips were harvested on the buccal plates of the missing tooth. An infrared camera and an implantable thermocouple collaboratively monitored in vivo real-time bone temperature at the drilling sites. In vitro performances of cells from bone chips, including cell number, viability, proliferation, migration, ALP activity, in vitro mineralization, mRNA transcriptional level of osteogenic genes and heat shock protein 70 (HSP-70), and HSP-70 expression at the protein level were also studied.

**Results:**

500 rpm produced mild local hyperthermia with a 2–6 °C temperature rise both on the cortical surface and inside the cortical bone. It also held comparable or enhanced cell performances such as cell number, viability, proliferation, migration, ALP activity, in vitro mineralization, and osteogenic genes expression.

**Conclusions:**

In-situ non-irrigated higher-speed drilling at 500 rpm using a screw drill is versatile, efficient, and thermal friendly and improves the bio-quality of bone chips. Our novel technique holds clinical translational potential in GBR application.

**Supplementary Information:**

The online version contains supplementary material available at 10.1186/s12903-023-03611-w.

## Background

Over 50% of dental implant surgeries have involved the use of bone grafts [1]. Among them, autograft has been broadly recognized as the gold standard material for GBR [2, 3]. It is pivotal to efficiently harvest bone autografts with high osteogenic quality. Currently, the broadly-used non-irrigated low-speed drilling technique has intrinsic shortcomings for bone autograft harvest [4]. Although debatable, cortical perforation is still recommended on GBR sites, especially on mandibular sites with thick buccal plate, to facilitate blood supply and angiogenesis [5]. However, low-speed drilling usually uses implant drills which are unsuitable for buccal cortical perforation since they are too large in diameter. Besides, it may be inappropriate in GBR surgeries without implant placement and implant drills. Moreover, it also has difficulty harvesting bone chips efficiently on dense-bone sites.

A facile solution is to increase the drilling speed. Nevertheless, high-speed drilling may directly increase thermal stress on the surrounding bone [6, 7]. Meanwhile, it can even cause irreversible thermal osteonecrosis, resulting in screw/implant loosening, crippled osteogenic capacity, and bone grafting failure [7, 8]. Generally, 47 °C for 1 min is accepted as the threshold for the occurrence of thermal osteonecrosis [9]. Within what speed range can non-irrigated higher-speed drilling still be bio-safe to bone remains unknown.

Interestingly, the effects of temperature elevation could be double-edged. Mild local hyperthermia (MLH) could also promote osteogenesis [10–12]. Heat shock proteins (HSPs) are a family of stress-induced proteins synthesized in response to diverse stimuli, including heat [13]. Among them, HSP-70 has been reported to play a role in MLH-based bone regeneration [14]. However, the role of HSPs (HSP-70) in non-irrigated higher-speed bone drilling has not been studied.

Currently, most thermal assessments of bone drilling involve the non-vital models including theoretical models, resin blocks, and *ex-vivo* bone blocks [15–18]. Such results could be of less value for clinical translational research due to the huge divergence between these models and the *in-vivo* clinical scenario. Only a few studies have investigated the in vivo temperature change during bone drilling [19]. Studies on autograft quality were mainly conducted at low drilling speeds (45–200 rpm, with 50 rpm being the most common choice) [20]. Moreover, these studies collected bone chips simply from implant osteotomy cavity, unable to achieve buccal cortical perforation. Consensus on the optimal drilling speed for bone autograft harvest has not been reached yet. Knowledge about in vivo thermal reactions of bone under non-irrigated higher-speed drilling and its influences on autograft quality is still lacking.

To address the above issues, we proposed a novel approach to efficiently harvesting bone autografts via in-situ non-irrigated higher-speed drilling with simultaneous cortical perforation. To our best knowledge, this is the first preclinical animal study to investigate the in vivo real-time thermal profile of surrounding bone and the in vitro bio-quality of bone chips using such a technique. We hypothesize that increasing the drilling speed within a certain range will incur no thermal damage and even enhance the osteogenic performances of autografts. This study sheds light on clinical translational research for the technical optimization of bone autografts harvest technique in GBR application.

## Methods

### Animals and group design

Eight male beagle dogs (3–4 years old, 18–20 kg, with complete permanent dentition, with good dental/general conditions) were used in this study. Animals were raised individually in standard kennel cages in an area with the same veterinary care and free access to food and water. Dogs were housed one week prior to surgery for adaptation, and were fasted overnight before surgery to prevent vomiting.

Animal experiments comprised two stages: stage I, extraction of the third mandibular premolars to create 16 partially edentulous sites (left and right) to simulate the tooth-missing situation; stage II, bone autografts harvest and real-time thermographic assessments on the edentulous sites after three months. Sixteen edentulous sites (left and right) were randomly allocated into four non-irrigated treatment groups using a random digit table method: (A) manual chisel (HU-FRIEDY, US); (B) drilling at 50 rpm; (C) drilling at 500 rpm; (D) drilling at 1000 rpm. Bone drilling in groups B-C-D was conducted with a retaining screw drill (HN, Korea; diameter: 1 mm).

### Surgical procedures

Surgical operations were all conducted under general anesthesia using pentobarbital sodium (30 mg/kg, intravenous injection). The dog was side-laid on the table with the surgical field facing upward. An assistant helped to stabilize its head during surgery. In stage I, the third mandibular premolars of both sides were carefully extracted with forceps, with the remaining bony buccal plates intact. In stage II, trapezoidal mucoperiosteal flaps were elevated from the second to the fourth premolars to expose the buccal plate for bone autografts collection. Penicillin (800,000 U/day) was used for three days after surgery.

### Bone autografts harvest

**Fig. 1:**
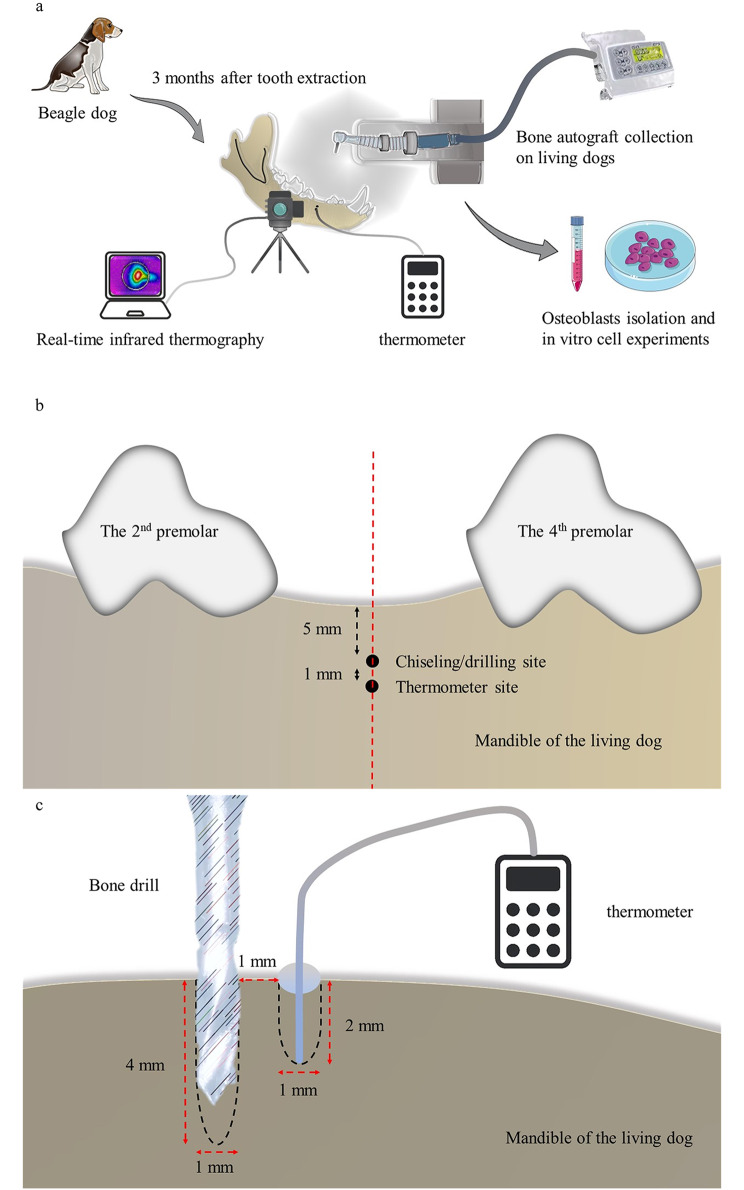
Schematic illustration. (**a**) A schematic illustration of the research process. (**b**) The drilling and thermocouple sites from the mesiodistal direction. (**c**) The drilling and thermocouple sites from the buccolingual direction (along the red dotted line in Fig. 1b)

Bone chips were harvested on the buccal plate of the missing third premolars. Group A collected bone chips by chiseling on the cortical surface, and group B-C-D harvested the in-situ bone chips piling up around the cortical perforation holes. Bone drilling in Group B-C-D was performed using a fixed apparatus (Fig. 1a) which mainly included a drill head and a supporting bed. Specifically, the drilling sites were located on the middle line of the missing tooth 5 mm below the alveolar ridge (Fig. 1b). Drilling was conducted under the same parameters (depth, 4 mm; torque, 35 Ncm; recording time, 10 s).

The harvested bone chips were temporarily stored in sterile tubes containing 2 mL PBS and were immediately transferred to the lab in 15 min. Tubes were all weighed before and after adding bone chips.

### In vivo thermographic assessments

In vivo real-time temperature change and spatial distribution during drilling were recorded with a type K thermocouple (Taishi, Taiwan) for the intra-cortical bone temperature and an infrared camera (Fotric 226s, macro lens M100) for the temperature of the cortical bone surface. Briefly, a hole for the thermocouple was pre-created at 1 mm apically from the harvesting hole with a depth of 2 mm (Fig. 1b-c). The hole was sealed with a thermally conductive paste to minimize heat loss. The tripod-supported infrared camera was fixed to keep 10 cm away from the surgical site. The temperature on the cortical surface was measured with the thermocouple to calibrate the camera.

The initial temperature, final temperature, and temperature changes were recorded as T0, T10, and ΔT10, respectively. It has been confirmed that MLH with 2–6 °C higher than body temperature (37 °C) could promote bone regeneration, and 47 °C has been broadly recognized as the threshold for thermal osteonecrosis [9–11]. Based on that, we categorized the temperature elevation range into four types: thermal fluctuation type (TF, < 2 °C); mild local hyperthermia type (MLH, 2–6 °C); thermal damage type (TD, 6–10 °C); thermal osteonecrosis type (TO, > 10 °C). AnalyzIR software (FOTRIC, Shanghai) extracted thermal data from thermographic images. A 5 × 5 mm^2^ area centered on the drilling site was chosen as the volume of interest. Thermal tests (n = 4) were all conducted in an air-conditioned operation room at 30.0 °C.

### Primary cell isolation and cell culture

Bone samples were gently washed with PBS three times and cultured in α-MEM, supplemented with 100 U/ml penicillin-G, 100 mg/ml streptomycin, and 15% fetal bovine serum (Baoxin, China) under 5% CO_2_ at 37 °C. The medium was refreshed every three days. Images were captured under a microscope to observe the morphology of cells on days 5, 10, and 14. The outgrown cells from bone chips were quantified per gram of wet sample after two weeks (n = 4).

Cells were passaged at 80% confluency and expanded for use at passages 3–4 except for the trypan blue staining, which used primary cells. Cells were cultured in 96-well plates at 1000/well for cell proliferation assay, in 6-well plates at 10,000/well for ALP staining and quantitative real-time polymerase chain reaction (RT-qPCR) assays, and in 6-well plates at 500,000/well for cell migration assay.

### Cell viability

The viability of primary cells on day three was studied by trypan blue staining (Solarbio, China) following the manufacturer’s protocol. Dead cells in blue-stained balloon morphology were captured under a microscope, and the live/dead cell ratio was quantified by cell counting (n = 4).

### Cell proliferation

Cell counting kit 8 (Biyuntian, China) assay was performed on days 1, 3, 5, and 7 as per the manufacturer’s protocol (n = 4). Absorbance at 450 nm was read, with day 1 being the reference line.

### Cell migration

Briefly, the cell monolayer was scratched by a 200-*µ*L pipette tip to create a wound with standard width. Wounded cells were washed off, and serum-free α-MEM returned to the remaining cells. Microscopic images were captured at 0, 12, and 48 h, and healing rates were quantified by calculating the wound shrinkage relative to 0 h using ImageJ software (National Institutes of Health, USA) (n = 4).

### ALP staining and activity

Cells were induced with osteogenic supplements (10 nmol/L dexamethasones, 50 *µ*g/mL ascorbic acid, and 5 mmol/L β-glycerophosphate) for one week. ALP staining (Solarbio, China) and ALP activity (Nanjing Jiancheng, China) were performed following the manufacturer’s instructions (n = 4). Absorbance at 510 nm was measured.

### Alizarin red staining

To detect in vitro mineralization of cells, alizarin red staining (Sigma, USA) was performed following the manufacturer’s protocol after osteogenic induction for 21 days (n = 4). Incubated with 100 mM cetylpyridinium chloride (Sigma) for 30 min, calcium accumulation of samples was quantified by measuring the OD values at 560 nm. The mean ODs of blank control were subtracted from the ODs of all test groups (see supplementary material S1).

### RT-qPCR

PCR was conducted to check the in vitro osteogenic capability and the *HSP-70* expression of cells. After one week of osteoinduction, total RNA was extracted using Trizol (Invitrogen, USA). PCR (n = 4) was performed using a SYBR Premix Ex Taq (Takara, Shiga, Japan) according to the manufacturer’s protocol. Relative mRNA transcription levels of bone morphology protein 2 (*BMP-2*), osteocalcin (*OCN*), collagen 1 (*Col-1*), and HSP-70 were studied. Primer sequences are as follows: *BMP-2* forward: CCAAGGCGTGCTGTGTACCAA; *BMP-2* reverse: ACCCACAACCCTCCACAACCA; *OCN* forward: AGAGGTGGTGCAGCCTTCGT; *OCN* reverse: GTCAGCCAGCTCGTCACAGTTG; *Col-1* forward: AGTGGTTTGGATGGTGCCAA; *Col-1* reverse: TCCATTTTCACCGGGGCTAC; *HSP-70* forward: CCGAAGAGAAGAGACCGAGC; *HSP-70* reverse: CTCAGGCTCACGTTCAGGTT; *GAPDH* forward: TCCATCTTCCAGGAGCGAGA; *GAPDH* reverse: CTCCATGGTGGTGAAGACCC.

### Western blot

Briefly, proteins were extracted using RIPA buffer (Beyotime), quantified by BCA assay (Solarbio), separated on 12% SDS-PAGE, and transferred onto a PVDF membrane (Millipore, USA). Primary antibodies against GAPDH and HSP-70 were then added for incubation overnight at 4 °C. The membrane was treated with the secondary antibody for 4 h and visualized using chemiluminescent reagents (Beyotime) according to the manufacturer’s instructions. Antibodies included Anti-GAPDH (1:10000, ab8245, Abcam), Anti-Hsp70 (1:1000, ab2787, Abcam), and HRP Conjugated AffiniPure Goat Anti-mouse IgG (H + L) (1:10000, BA1051, Boster). Protein abundance was quantified by measuring the gray value of blots in scanned images using ImageJ software (see supplementary material S2).

### Statistical analysis

Data involved at least three independent measurements and were analyzed using One-way ANOVA with Bonferroni’s multiple comparison test by GraphPad Prism 8.3. Results were presented as mean ± standard deviation. The significance level was set at p < 0.05.

## Results

### In vivo thermal assessments on the cortical surface

**Fig. 2:**
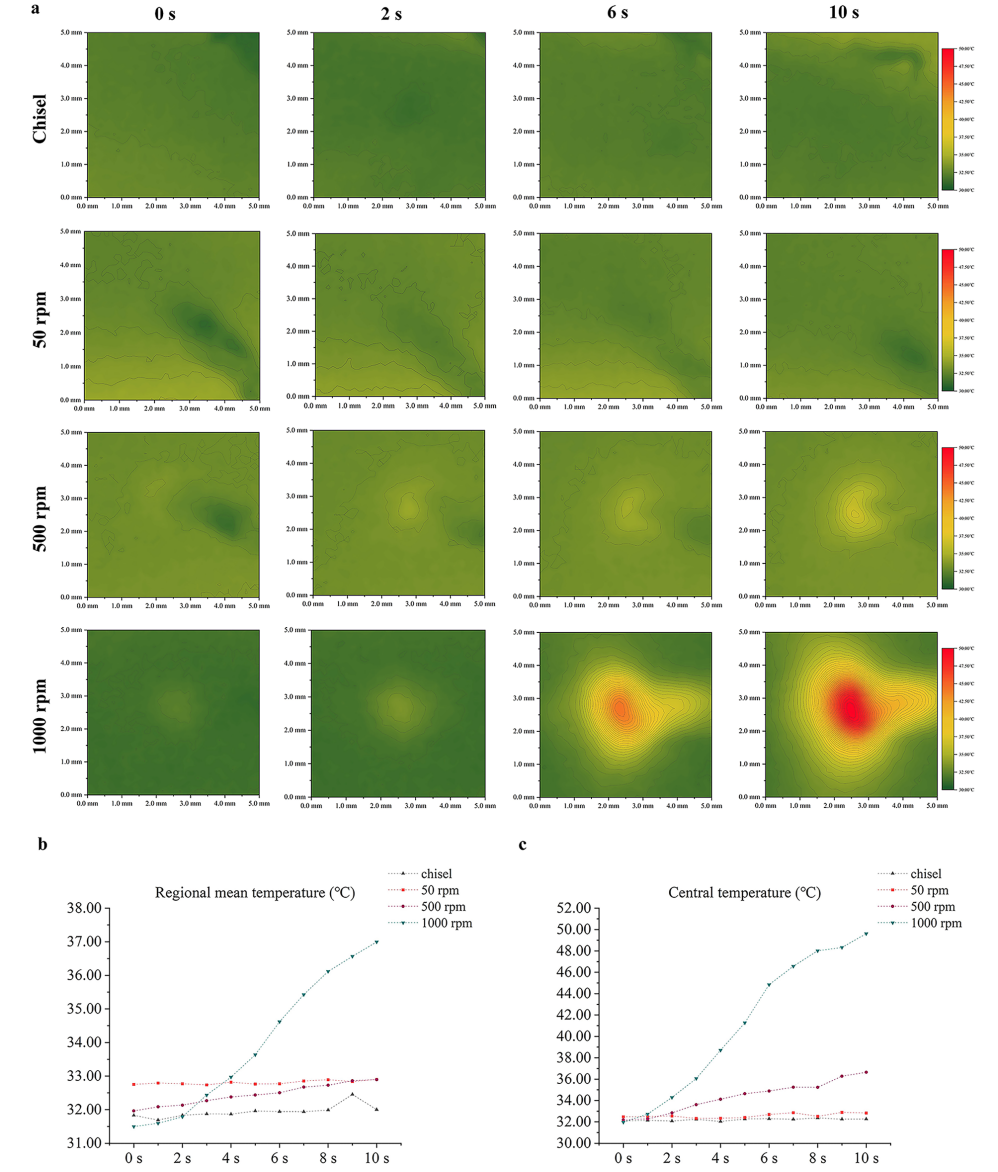
In vivo thermal assessments on the cortical surface. (**a**) Real-time temperature distribution on the cortical surface. (**b**) regional mean temperature-time curve within 10 s. (**c**) Central temperature-time curve within 10 s

Figure 2a depicts the real-time temperature distribution in a 5 × 5 mm^2^ square centered on the drilling site, with red representing higher temperature. Chisel and 50 rpm bore no noticeable change, 500 rpm showed mild temperature elevation, and 1000 rpm presented a large area of dramatic temperature rise at the central part at T10 (Fig. 2a). Regional mean temperature surged at 2 s in group 1000 rpm and fluctuated within 32.00–33.00 °C in the other three groups. The central temperature in the chisel and 50 rpm groups fluctuated around 32.00 °C, gradually climbed to 36.60 °C in 500 rpm, and surged to almost 50.00 °C in 1000 rpm at T10 (Fig. 2b-c).

### In vivo temperature distribution on the cortical surface at T10

**Fig. 3:**
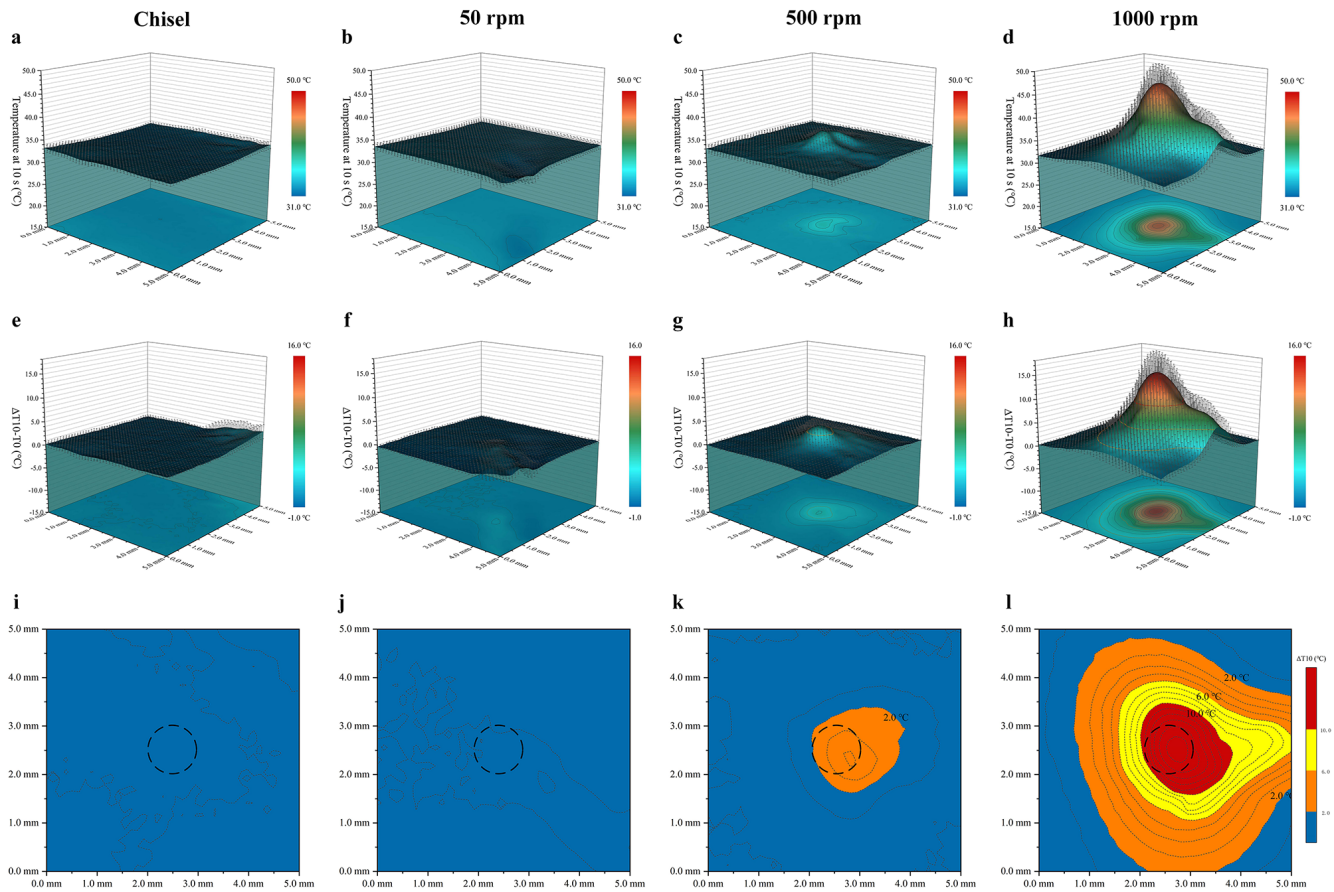
In vivo temperature distribution on the cortical surface at T10. (**a-d**) 3D temperature distribution on the cortical surface at T10. (**e-h**) 3D temperature elevation on the cortical surface at T10. (**i-l**) Areas of different types of temperature elevation at T10 on the cortical surface. The black dotted circles in Fig. 3i-l represent the central drilling sites (1 mm in diameter). Blue, orange, yellow, and red represent the TF, MLH, TD, and TO types respectively

3D temperature distribution on the cortical surface at T10 was presented in Fig. 3. Chisel and 50 rpm manifested as a flat plain, 500 rpm was small hill-like in the center, while 1000 rpm presented as a substantial mountain-like heated area in the central part (Fig. 3a-d). ΔT10 suggested that the central part presented higher temperature elevation with a negative relation between temperature elevation and the distance to the center (Fig. 3e-h). The temperature elevation types at the central region were TF type in chisel and 50 rpm, MLH type in 500 rpm, and TO type in 1000 rpm, respectively (Fig. 3i-l). MLH region in 500 rpm covers an area of 1.5 × 2 mm, and the TD/TO region in 1000 rpm covers an area of 3 × 3.5 mm.

Central temperatures at T10 were 32.26 ± 0.32, 32.83 ± 0.78, 36.64 ± 0.44, and 49.62 ± 1.68 °C in chisel, 50 rpm, 500 rpm, and 1000 rpm, respectively (p < 0.05). ΔT10 were 0.16 ± 0.36, 0.36 ± 0.29, 4.46 ± 1.53, and 17.65 ± 1.91 °C in those groups, respectively (p < 0.05) (Table 1).

**Table 1:** In vivo central temperature of the cortical surface at T10

Groups	Temperature (°C)/ Temperature elevation type	Overall comparison (*P-value*)	Multi-comparisons (P value)
T10			
A、chisel	32.26 ± 0.32	*< 0.000*(*)	A-B: *> 0.999*(ns)
B、50 rpm	32.83 ± 0.78	ANOVA	B-C: *0.001*(*)
C、500 rpm	36.64 ± 0.44		C-D: *< 0.000*(*)
D、1000 rpm	49.62 ± 1.68		Bonferroni test
ΔT10			
A、chisel	0.16 ± 0.36 TF	*< 0.000*(*)	A-B: *> 0.999*(ns)
B、50 rpm	0.36 ± 0.29 TF	ANOVA	B-C: *0.003*(*)
C、500 rpm	4.46 ± 1.53 MLH		C-D: *< 0.000*(*)
D、1000 rpm	17.65 ± 1.91 TO		Bonferroni test

ns, non-significant; *, significant

### In vivo thermal assessments of intra-cortical bone at T10

Intra-cortical temperatures at 1 mm away from the drilling hole at T10 were 33.50 ± 0.26, 35.15 ± 0.21, 37.85 ± 0.37, and 50.33 ± 0.15 °C in chisel, 50 rpm, 500 rpm, and 1000 rpm, respectively (p < 0.05). ΔT10 were 0.28 ± 0.22, 1.90 ± 0.34, 5.18 ± 0.33, and 17.38 ± 0.61 °C in those groups, respectively (p < 0.05) (Table 2).

**Table 2:** In vivo intra-cortical bone temperature at T10

Groups	Temperature (°C)/ Temperature elevation type	Overall comparison (*P-value*)	Multi-comparisons (P value)
T10			
A、chisel	33.50 ± 0.26	< 0.000(*)	A-B: *< 0.000*(*)
B、50 rpm	35.15 ± 0.21	ANOVA	B-C: *< 0.000*(*)
C、500 rpm	37.85 ± 0.37		C-D: *< 0.000*(*)
D、1000 rpm	50.33 ± 0.15		Bonferroni test
ΔT10			
A、chisel	0.28 ± 0.22 TF	*< 0.000*(*)	A-B: *0.001*(*)
B、50 rpm	1.90 ± 0.34 TF	ANOVA	B-C: *< 0.000*(*)
C、500 rpm	5.18 ± 0.33 MLH		C-D: *< 0.000*(*)
D、1000 rpm	17.38 ± 0.61 TO		Bonferroni test

ns, non-significant; *, significant

### Cell outgrowth

**Fig. 4:**
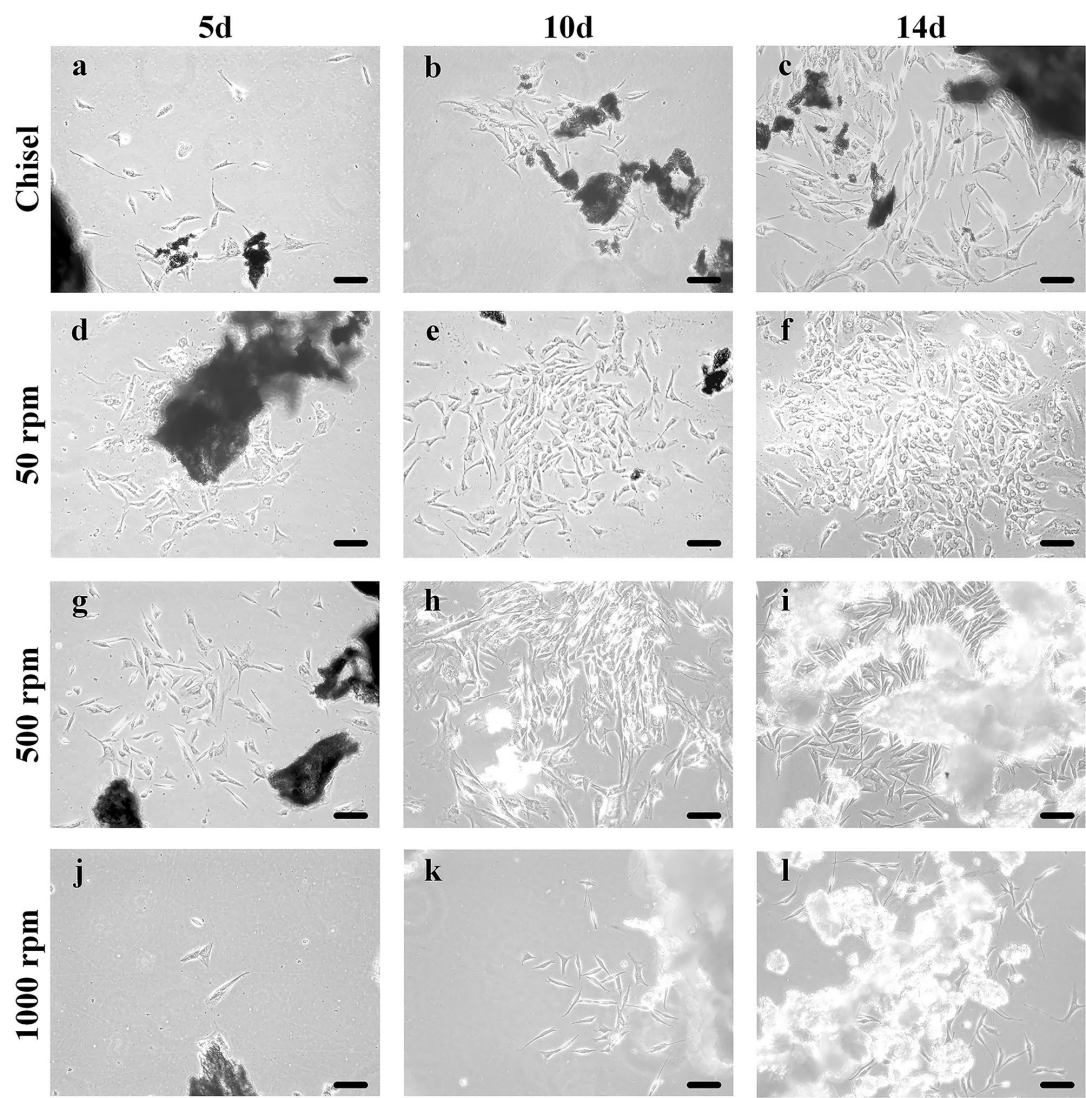
Cell outgrowth from bone chips at day 5, 10, 14. (**a-c**) chisel. (**d-f**) 50 rpm. (**g-i**) 500 rpm. (**j-l**) 1000 rpm. bar = 50 *µ*m

Few cells on day 5 and a few on day 14 could be seen in group 1000 rpm. Numerous cells with typical spindle-like osteoblast morphology grew from bone chips on day 14 in group chisel, 50 and 500 rpm (Fig. 4a-l).

### Cell counting, viability, and proliferation

**Fig. 5:**
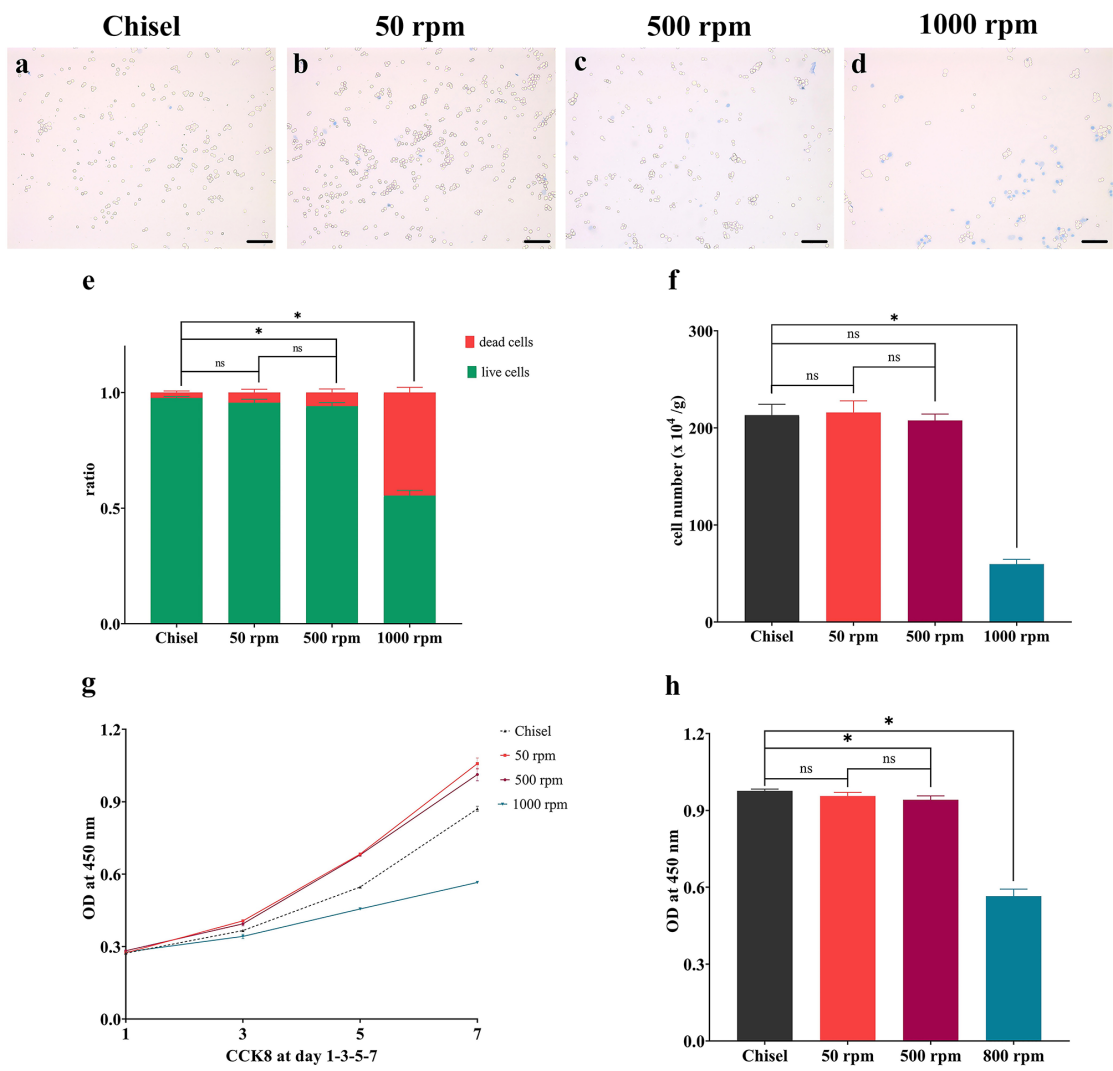
Cell counting, viability, and proliferation. (**a-d**) Trypan blue staining at day 3. (**e**) Living/dead cell ratio at day 3. (**f**) Primary cell counting at day 14. (**g**) Cell proliferation at day 1, 3, 5, and 7. (**h**) Cell proliferation at day 7. bar = 50 *µ*m; ns, non-significant; *, significant

Living cells were dominant in chisel, 50 rpm, and 500 rpm, while a large number of dead cells (blue-stained) appeared in group 1000 rpm (Fig. 5a-d). Living cell rates in the above groups were 97.65 ± 0.68%, 95.64 ± 1.44%, 94.13 ± 1.56%, and 56.54 ± 2.75%, respectively (p < 0.05) (Fig. 5e). Primary cell counting was 213.00 ± 11.27, 215.80 ± 12.03, 207.60 ± 6.65, and 59.60 ± 4.93 (x 10^4^ per gram of bone chips), respectively (p < 0.05) (Fig. 5f). 50 rpm and 500 rpm proliferated much faster than the others, and group 1000 rpm was the slowest (p < 0.05) (Fig. 5g-h). No statistical significances were detected between 500 rpm and 50 rpm regarding living cell rate, cell counting, and cell proliferation at day 14 (p > 0.05).

### Cell migration and ALP activity

**Fig. 6:**
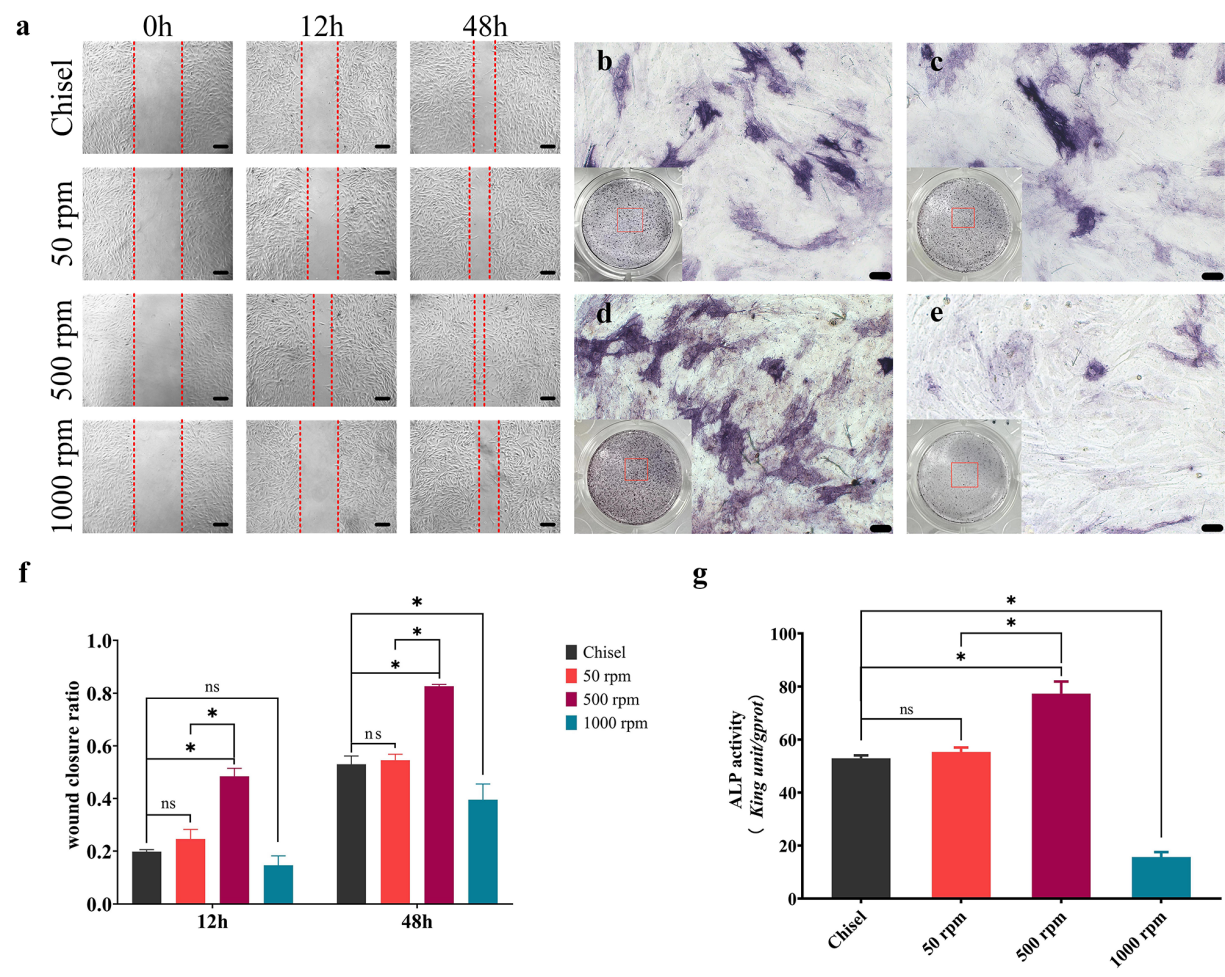
Cell migration and ALP activity. (**a**) Wound healing at 0, 12, and 48 h. (**b-e**) ALP staining of chisel, 50 rpm, 500 rpm, and 1000 rpm, respectively. (**b-e**) were magnified regions captured from red squares in general views. (**f**) Wound healing rate at 12 and 48 h. (**g**) ALP activity. bar = 50 *µ*m; ns, non-significant; *, significant

The wound healed similarly in group chisel and 50 rpm, while 500 rpm and 1000 rpm showed the fastest and slowest healing speed, respectively (Fig. 6a). 500 rpm showed the deepest ALP staining, while only a few stained cells could be seen in 1000 rpm (Fig. 6b-e). Healing rates were 53.01 ± 3.13%, 54.58 ± 2.23%, 82.63 ± 0.67%, and 39.55% ± 5.94% in chisel, 50, 500, 1000 rpm, respectively (Fig. 6f). Quantitative results of ALP activity were 53.00 ± 1.04, 55.33 ± 1.68, 77.35 ± 4.56, and 15.61 ± 1.91 king unit/*gprot* in the above groups, respectively (Fig. 6g). Comparisons between 500 rpm and the other groups held statistical significance (p < 0.05).

### Alizarin red staining

Staining was strongest in 500 rpm and weakest in 1000 rpm (Figure S1a-d). Quantitative results of calcium accumulation were 2.89 ± 0.05, 2.91 ± 0.08, 6.42 ± 0.04, and 1.69 ± 0.02 in the above groups, respectively (Figure S1e). Comparisons between 500 rpm and the other groups held statistical significance (p < 0.05).

### RT-qPCR

**Fig. 7:**
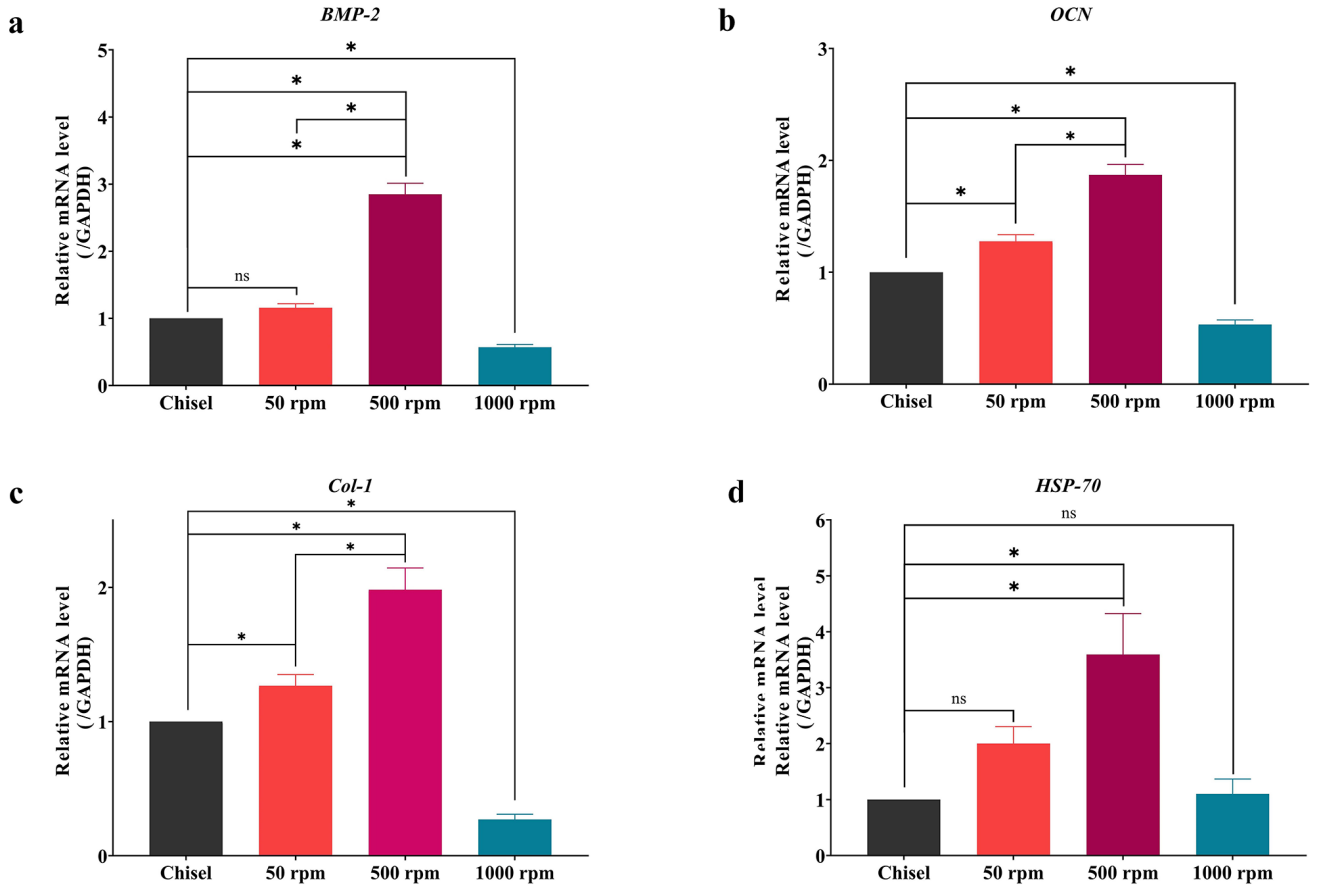
RT-qPCR. (**a**) *BMP-2*. (**b**) *OCN*. (**c**) *Col-1*. (**d**) *HSP-70*. ns, non-significant; *, significant

Compared with chisel, the mRNA transcription level of *BMP-2* in 50, 500, and 1000 rpm increased to 1.16 ± 0.06, 2.85 ± 0.16, and 0.57 ± 0.04 folds, respectively (chisel-50 rpm: p > 0.05; other comparisons: p < 0.05); *OCN* increased to 1.28 ± 0.06, 1.87 ± 0.09, and 0.53 ± 0.04 folds, respectively (p < 0.05); *Col-1* increased to 1.27 ± 0.08, 1.98 ± 0.16, and 0.27 ± 0.04 folds, respectively (p < 0.05); *HSP-70* increased to 2.00 ± 0.30, 3.59 ± 0.73, and 1.10 ± 0.27 folds, respectively (chisel-50 rpm: p > 0.05; chisel-1000 rpm: p > 0.05; other comparisons: p < 0.05) (Fig. 7a-d).

### Western blot

HSP-70 expression of 500 rpm was significantly higher than that of other groups (Figure S2a). With the chisel group being a control, relative protein expression levels were 1.00 ± 0.19, 1.10 ± 0.19, 2.09 ± 0.32, and 0.54 ± 0.04 in chisel, 50, 500, and 1000 rpm, respectively (Figure S2b). Comparisons between 500 rpm and the other groups held statistical significance (p < 0.05).

## Discussion

The osteogenic quality of the harvested bone autografts mainly comes from minerals, viable cells, the embedded bio-factors, and the bio-factors that cells secrete after transplantation [3, 21]. Technically, such bio-quality highly relies on how these bone chips are obtained [22–26]. Various factors, such as peak temperature, spatial temperature distribution, and the composition of bone chips, may have a joint impact on the bio-quality of bone chips during surgery.

Temperature profile is of tremendous biological and clinical significance. Thermal osteonecrosis may appear as the peak temperature exceeded the critical value (47 °C) in group 1000 rpm. High-speed drilling at 1000 rpm may cause enormous thermal damage to cells, which has been confirmed in some studies that high-speed drilling could result in empty osteocyte lacuna and the lack of osteoblasts and lining cells [4, 27]. Thermal osteonecrosis could further lead to implant/screw loosening, bone healing problems, and grafting failure [8, 9]. Our results showed that temperature elevation was positively related to drilling speed, consistent with many previous work [28–30]. This fact could be explained by the increased drill-bone contact number, bone chips accumulation, and friction [15]. On the other hand, increasing the speed could also reduce heat production partially by shortening operation time [31]. Currently, consensus on the optimal speed range for non-irrigated drilling is still lacking. Our results suggested that drilling at less than 500 rpm should be thermally safe. Interestingly, 500 rpm in this study produced more heat than some studies with similar speeds (non-irrigated 462 rpm, 35.2–43.0 °C) [32], which may be explained by higher baseline temperature and different animals adopted in our study.

More academic attention should be put on the spatial distribution of temperature. Heat accumulates around the drilling hole and then dissipates in a distance-based pattern. Bone drilling at 1000 rpm could produce an overheated area at 0-0.5 mm away from the hole [33]. Non-irrigated high-speed drilling could result in thermal osteonecrosis at 6 mm (2000 rpm) or 9.5 mm (3000 rpm) away from the drilling site, and thermal risk in the horizontal direction was higher than in the vertical direction [16, 34]. Nevertheless, those studies were all based on theoretical or in vitro models rather than the in vivo real-time thermographic assessment in this study. Cortical bone suffered higher temperature rise during drilling due to thermal conductivity differences and longer drill-bone contact [30, 35]. In contrast, our results showed that the temperature of the intra-cortical bone (vertical direction) was higher than that of the cortical surface (horizontal direction) at T10. Reasons for such inconsistency could be: (1) The dense mandible bone resulted in more mechanical friction. (2) Poor blood circulation of cortical plate failed to provide enough heat dissipation. (3) The interfaces of heat dissipation were the bone-air type on the cortical surface and the bone-drill type inside the hole respectively, with the former type undergoing active heat exchange. In fact, we measured the intra-cortical temperature at 1 mm away from the perforation hole, so the actual in-situ temperature inside the perforation hole should be even higher.

The spatial temperature distribution is of immense clinical significance. Temperature distribution along the cortical surface can influence the health condition of bone around the drilling holes; temperature distribution inside the holes can directly affect the bio-quality of the harvested bone chips. In this study, 500 rpm did not produce TD and TO types of temperature elevation. Instead, it behaved as MLH type both on the cortical surface (a 1.5 × 2 mm area) and inside the cortical bone (1 mm away from the hole, 2 mm in depth). Since MLH has been broadly reported to enhance bone regeneration [11, 12], the bone tissue in the above regions in group 500 rpm could benefit from the MLH-based osteogenesis promotion. Per contra, 1000 rpm produced a 3 × 3.5 mm TD/TO region, suggesting that bone tissue at 2-2.5 mm from the hole (1 mm in diameter) may suffer from quality damage/loss. Clinicians should be aware of the benefited and compromised areas during surgery at different drilling speeds.

The up-regulation of HSP-70 helped further explain why MLH improved the osteogenic performances of group 500 rpm. Wisdom of heat-induced bone regeneration abounds: MLH facilitated stem cell proliferation, ALP activity, in vitro mineralization, and improved osteogenic differentiation via HSPs [10]; animal experiments have further proved that MLH (40–43 °C, 41–42 °C) could promote in vivo bone repair possibly due to the upregulation of HSPs [11, 12]. Additionally, HSP-70 has been reported to improve osteogenic activities, possibly through the ERK or Wnt/β-catenin pathway [36, 37]. Our results revealed that HSP-70 was upregulated in the MLH environment in group 500 rpm, consistent with some previous study [14]. Interestingly, we still successfully cultured a handful of primary osteoblast-like cells in 1000 rpm. It could be explained by the short operation time (10 s) which was not enough to inactivate all the embedded cells. Temperature threshold is also called the cumulative equivalent minutes [9, 38]. Thermal damage of bone is influenced both by the extent of temperature elevation and its duration.

Lastly, the composition of bone chips also affected their osteogenic quality. In this study, chisels harvested cortical chips on the bone surface, and the other three drilling techniques can obtain both cortical and cancellous contents since their drilling depth was deep enough to reach bone marrow [39]. Cancellous bone is generally preferable in GBR because of higher cellularity and surface area for re-vascularization [40]. Differently, cortical bone contains abundant BMP-2 and is more resorption-resistant [41]. This may help explain why 50 rpm slightly outperformed the chisel in osteogenic properties. Less cultured cells in 500 rpm compared with group chisel and 50 rpm (non-significant) may result from the slight mechanical damage of higher drilling speed. To address different clinical situations, an appropriate combination of the two bone types should theoretically achieve optimal grafting outcomes [42, 43]. For minor bone defects where autogenous bone chips are adopted alone or used with other bone substitutes, cancellous bone is more favored; for more significant bone defects which should be stably reconstructed, more cortical bone or even on-lay cortical blocks should be considered.

In summary, quality of bone chips in this study was influenced by multiple factors, including peak temperature, spatial temperature distribution, and the composition of bone chips. Non-irrigated drilling can obtain favorable cortical-cancellous bone contents, and 1000 rpm caused much thermal damage, while 500 rpm could provide the MLH-based osteogenic promotion. Increasing the drilling speed could improve the efficiency of bone drilling and autograft collection. However, meanwhile, it can incur non-negligible thermal damage or even thermal osteonecrosis in the non-irrigated scenario. Therefore, clinicians must focus on the underlying thermal damage when adopting higher drilling speed.

Regarding the limitations, it is unknown whether these data achieved by a particular screw drill in this study will still apply to other types of clinical drills. Besides, the conditions of host bone around the drilling hole remain unknown since no tissue stain for cell metabolism and bone turnover was conducted. Additionally, it is unclear how these bone autografts will behave in GBR application, and hence animal studies are imperative in the future to check the in vivo bone regeneration. Lastly, the beagles’ mandibles still present divergent physiological and thermal properties compared with the human jaw. Given the above situation, any data interpretation for human scenarios should be taken with caution.

## Conclusions

Designing a widely applicable technique to efficiently harvest bone autografts with simultaneous buccal cortical perforation is of clinical significance in dental bone grafting applications. Knowledge about the in vivo thermal reaction of bone tissue under non-irrigated higher-speed drilling and its influence on the bio-quality of autografts is still lacking. This study proposed and investigated a novel bone autograft harvest technique via non-irrigated in-situ higher-speed drilling using a particular screw drill in dogs for the first time. 500 rpm produced mild local hyperthermia without thermal damage and osteonecrosis, and it held comparable or even better osteogenic performances. The upregulation of HSP-70 may play a role in improving the bio-quality of bone chips.

Within the limitations of this study, with the advantages of higher efficiency, thermal safety, and enhanced osteogenic quality, non-irrigated in-situ drilling at 500 rpm with a screw drill shows clinical translational potential for bone autografts harvest in GBR application.

### Electronic supplementary material

Below is the link to the electronic supplementary material.

Supplementary Material 1

## Data Availability

The datasets used and/or analyzed during the current study are available from the corresponding author upon reasonable request.

## Abbreviations

HSP-70: Heat shock protein 70
GBR: Guided bone regeneration
MLH: Mild local hyperthermia
ALP: Alkaline phosphatase
RT-qPCR: Quantitative real-time polymerase chain reaction
BMP-2: Bone morphology protein 2
OCN: Osteocalcin
Col-1: Collagen 1
