# Supplementary material for: A novel technique to harvest bone autografts with mild local hyperthermia and enhanced osteogenic bone quality: a preclinical study in dogs
Source: BMC Oral Health. 2023 Nov 7;23:838. doi: 10.1186/s12903-023-03611-w (PMC10631188; doi:10.1186/s12903-023-03611-w)
Supplement: Supplementary file 1 — Supplementary Material 1 [file 12903_2023_3611_MOESM1_ESM.docx]

**A novel technique to harvest bone autografts with mild local hyperthermia and enhanced osteogenic bone quality: a preclinical study in dogs**

Tengfei Zhou^1^, Zekun Gan^1^, Hanfei Zhang^2^, Ziyi Liu^2^, Yiping Pu^3^*, Mingdeng Rong^1^*

1. Department of Periodontology and Oral Implantology, Stomatological Hospital, Southern Medical University, Guangzhou, 510280, China.
2. Stomatological Hospital, Southern Medical University, Guangzhou, 510280, China.
3. Department of Oral Surgery, Shanghai Ninth People's Hospital, College of Stomatology, Shanghai Jiao Tong University School of Medicine, Shanghai 200001, China.

* These two authors are considered as the joint corresponding authors.

**Figure S1. Alizarin red staining**


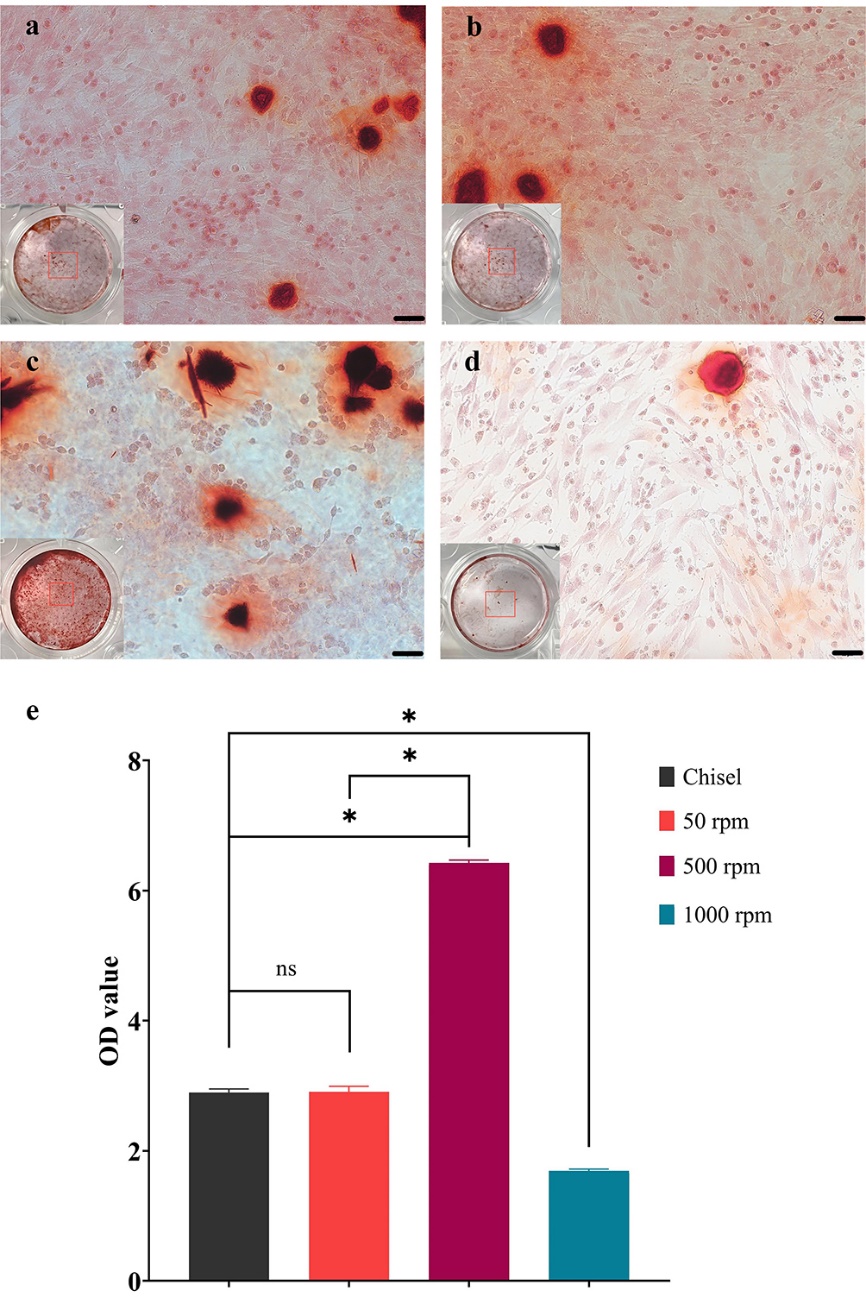


**Figure S1. Alizarin red staining.** a-d. Alizarin red staining of chisel, 50 rpm, 500 rpm, and 1000 rpm, respectively. a-d were magnified regions captured from red squares in general views. e. Quantitative analysis of calcium accumulation. bar = 50 *μ*m; ns, non-significant; *, significant.

**Figure S2. Western blot of HSP-70**


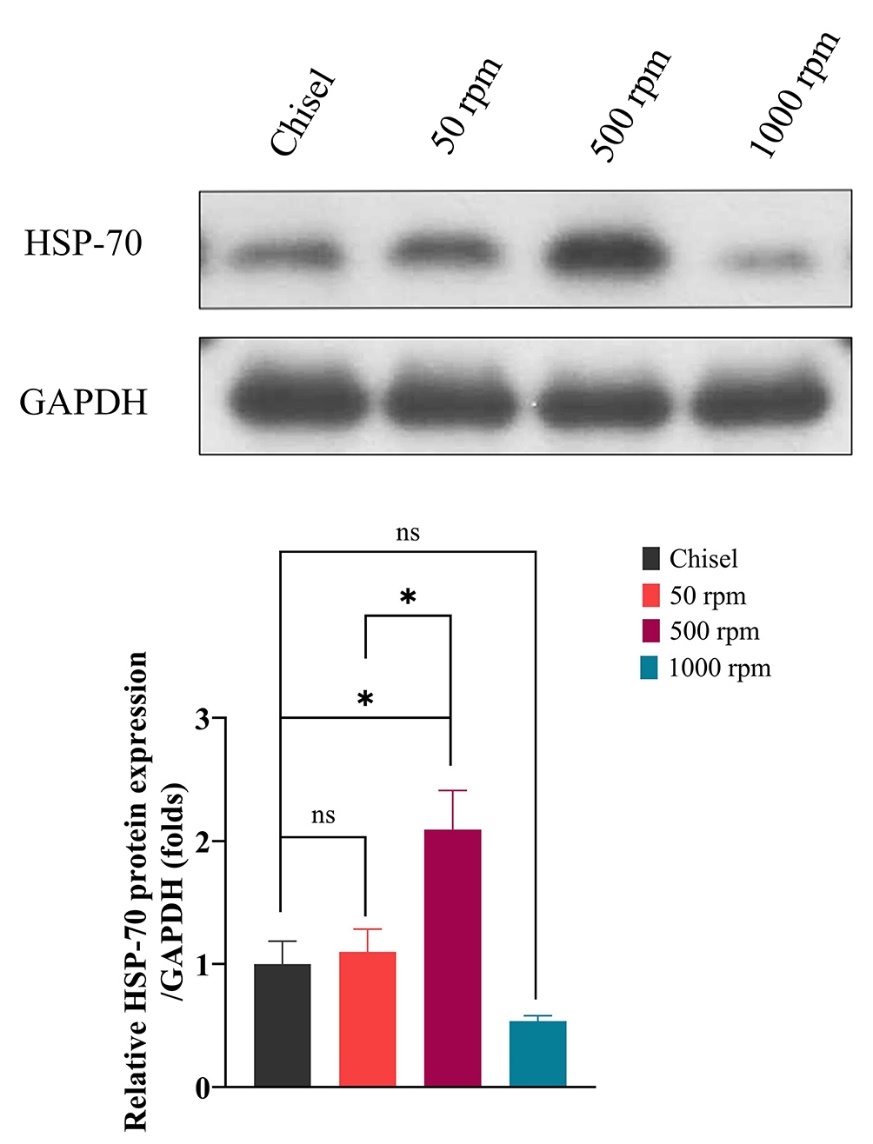


**Figure S2. Western blot of HSP-70.** a. Western blot of HSP-70 in chisel, 50 rpm, 500 rpm, and 1000 rpm, respectively. b. Relative protein expression level of HSP-70 normalized to GAPDH (chisel was set as the control group). ns, non-significant; *, significant.

**Corresponding author:** Mingdeng Rong

Department director

Department of Periodontology and Oral Implantology, Stomatological Hospital, Southern Medical University, Guangzhou, 510280, China

Phone number: +86 13710900791

Fax number: 34314299

Email: rmdeng@smu.edu.cn
